# Retraction: Role of T1 Ratio of Marrow, Diffusion-Weighted Imaging, and Chemical Shift Imaging in Multiparametric MRI of the Spine in Spinal Tuberculosis

**DOI:** 10.7759/cureus.r217

**Published:** 2026-01-22

**Authors:** Muhammad Fahad Idrees, Maryum Tayyab, Muhammad Haider, Maham Mujeeb, Usama Mazhar, Ahmad Nazir Tarar

**Affiliations:** 1 Department of Gariatrics, Walsall Manor Hospital, Walsall, GBR; 2 Department of Acute Medicine, Eastbourne District General Hospital, Eastbourne, GBR; 3 Department of Cardiology, Eastbourne District General Hospital, Eastbourne, GBR; 4 Acute Medical Unit, Walsall Manor Hospital, Walsall, GBR; 5 Department of Geriatrics, Stepping Hill Hospital, NHS Stockport Trust, Stockport, GBR; 6 Department of Internal Medicine, Manchester Royal Infirmary Hospital, Manchester University NHS Foundation Trust, Manchester, GBR

The Editor-in-Chief has retracted this article. An investigation by the journal found evidence of authorship manipulation. As a result, the journal no longer has confidence in the provenance and reliability of the article contents. The authors did not respond when contacted.

